# Response of Mustard Microgreens to Different Wavelengths and Durations of UV-A LEDs

**DOI:** 10.3389/fpls.2019.01153

**Published:** 2019-10-17

**Authors:** Aušra Brazaitytė, Akvilė Viršilė, Giedrė Samuolienė, Viktorija Vaštakaitė-Kairienė, Julė Jankauskienė, Jurga Miliauskienė, Algirdas Novičkovas, Pavelas Duchovskis

**Affiliations:** Institute of Horticulture, Lithuanian Research Centre for Agriculture and Forestry, Babtai, Lithuania

**Keywords:** light-emitting diodes, UV-A, growth, antioxidants, mineral elements, mustard microgreens

## Abstract

Ultraviolet A (UV-A) light-emitting diodes (LEDs) could serve as an effective tool for improving the content of health-promoting bioactive compounds in plants in controlled-environment agriculture (CEA) systems. The goal of this study was to investigate the effects of UV-A LEDs at different wavelengths (366, 390, and 402 nm) and durations (10 and 16 h) on the growth and phytochemical contents of mustard microgreens (*Brassica juncea* L. cv. “Red Lion”), when used as supplemental light to the main LED lighting system (with peak wavelengths of 447, 638, 665, and 731 nm). Plants were grown for 10 days under a total photon flux density (TPFD) of 300 µmol m^−2^ s^−1^ and 16-h light/8-h dark period. Different UV-A wavelengths and irradiance durations had varied effects on mustard microgreens. Supplemental UV-A radiation did not affect biomass accumulation; however, the longest UV-A wavelength (402 nm) increased the leaf area of mustard microgreens, regardless of the duration of irradiance. The concentration of the total phenolic content and α-tocopherol mostly increased under 402-nm UV-A, while that of nitrates increased under 366- and 390-nm UV-A at both radiance durations. The contents of lutein/zeaxanthin and β-carotene increased in response to the shortest UV-A wavelength (366 nm) at 10-h irradiance as well as longer UV-A wavelength (390 nm) at 16 h irradiance. The most positive effect on the accumulation of mineral elements, except iron, was observed under longer UV-A wavelengths at 16-h irradiance. Overall, these results suggest that properly composed UV-A LED parameters in LED lighting systems could improve the nutritional quality of mustard microgreens, without causing any adverse effects on plant growth.

## Introduction

Because of the rising awareness of health, people are changing their dietary habits and consuming more vegetables and herbs that are low in calories and an important source of minerals, vitamins, and other beneficial phytochemicals. Consequently, this has led to higher requirements for the nutritional quality of vegetables, which can be enhanced by the regulation of environmental and agronomic factors. Businesses are also seeking to extend the period in which they are able to supply consumers with fresh vegetables; therefore, their cultivation in controlled-environment agriculture (CEA) conditions is increasing (Bian et al., 2015; Dou et al., 2017; Rouphael et al., 2018). In addition to environmental factors, such as temperature, humidity, CO_2_, and nutrient concentrations, artificial lighting has a major effect on the quality of vegetables in CEA systems. Previously, traditional light sources such as fluorescent, high-pressure sodium (HPS), metal halide, and incandescent lamps have been used in CEA systems. However, these artificial light sources are not spectrally optimal or energy efficient. Nowadays, the use of energy-efficient light-emitting diode (LEDs) in CEA systems has many advantages, such as the ability to select light wavelengths, change light intensity, and reduce energy costs (Mitchell et al., 2015; Cocetta et al., 2017; Dou et al., 2017; Bantis et al., 2018). LED technology has been used to determine the effects of light quality and quantity on plant growth and development as well as to indicate the physiological response of plants to photooxidative changes by altering the production of phytochemicals such as anthocyanins, phenols, carotenoids, soluble sugars, and nitrates (Olle and Viršile, 2013; Carvalho and Folta, 2014; Ouzounis et al., 2015; Bian et al., 2015; Mitchell et al., 2015; Brazaitytė et al., 2016; Samuolienė et al., 2016; Bantis et al., 2018). It is well known that red and blue lights are absorbed by leaves better than other regions of the visible spectrum, and LEDs of such wavelengths are categorized as having the highest photon efficiency; therefore, the effect of red to blue light ratio on plants has been detailed in many studies (Massa et al., 2008; Olle and Viršile, 2013; Nelson and Bugbee, 2014; Mitchell et al., 2015; Bugbee, 2016).

The development of LED technology encourages a more detailed investigation of a wider light spectrum by incorporating green, far-red, and ultraviolet A (UV-A) LED wavelengths. The lack of UV radiation in CEA lighting systems adversely affects the nutritional quality of plants, especially those that are generally cultivated in open fields, mostly by decreasing the content of phenolic compounds (Iwai et al., 2010; Wargent and Jordan, 2013; Neugart and Schreiner, 2018). Although UV-A diodes in LED illumination could solve such problems, lower everyday applications of UV LEDs slowed their development and increased their cost because of the materials used in manufacturing processes. Moreover, UV LEDs have lower photon efficiency than other visible spectrum LEDs. Consequently, the usage of UV wavelengths in CEA is still in its initial stages (Wargent, 2016; Neugart and Schreiner, 2018). Although UV LED lighting is a new technology, research has been conducted on the effects of UV-A LED, as a part of different lighting systems or as a sole source of light, on the growth and metabolism of plants (Li and Kubota, 2009; Samuolienė et al., 2013; Khoshimkhujaev et al., 2014; Brazaitytė et al., 2015; Vaštakaitė et al., 2015; Lee et al., 2014; Brazaitytė et al., 2016; Goto et al., 2016; Rechner et al., 2017; Jensen et al., 2018). Some of these investigations showed that UV-A LED leads to higher total phenolic content, anthocyanin content, ascorbic acid concentration, and antiradical properties in leafy vegetables and herbs (Li and Kubota, 2009; Samuolienė et al., 2013; Brazaitytė et al., 2015; Vaštakaitė et al., 2015; Goto et al., 2016). However, these studies only investigated the effect of different UV-A wavelengths on plants, not the impact of the irradiance duration of a specific UV-A LED wavelength; the latter has not yet been studied.

*Brassica* vegetables are one of the most popular and widely grown vegetables in the world. These vegetables, including mustard (*Brassica juncea* L.), contain various health-promoting compounds such as carotenoids, chlorophylls, phenolic compounds, glucosinolates, and minerals and are an excellent source of fiber. Because of extensive plant breeding efforts, *Brassica* vegetables exhibit highly variable appearance, phytochemistry, and usage (Björkman et al., 2011; Frazie et al., 2017; Neugart et al., 2018). Nowadays, it is common to cultivate *Brassica* vegetables as microgreens. Microgreens are a non-traditional type of vegetables that are grown until the cotyledons have fully opened and first true leaves have fully emerged. Microgreens are harvested 1–3 weeks post-germination and exhibit a wide variety of flavors, colors, and textures, features that make them attractive ingredients in novel cuisine. Furthermore, microgreens contain higher amounts of bioactive compounds than seeds or mature plants (Xiao et al., 2012; Pinto et al., 2015; Kyriacou et al., 2016; Kyriacou et al., 2017). CEA allows year-round harvesting of microgreens and the manipulation of light quantity and quality to alter nutritional attributes of plants (Samuolienė et al., 2012; Brazaitytė et al., 2016; Kyriacou et al., 2016; Kyriacou et al., 2017). In this study, we aimed to investigate the effects of different UV-A LED wavelengths (366, 390, and 402 nm) at two different durations (10 and 16 h) on the growth and phytochemical composition of mustard microgreens, when used in addition to the solid-state LED lighting system.

## Materials and Methods

### Plant Material and Growth Conditions

Mustard microgreens (*B. juncea* L. cv. “Red Lion”) were grown in a peat substrate (Profi 1, Durpeta, Lithuania) (pH 5–6) in 0.5-L plastic containers (18 cm × 11 cm × 6 cm) for 10 days. The nutritional composition (mg L^−1^) of the substrate was as follows: nitrogen (N), 110; phosphorous pentoxide (P_2_O_5_), 50; potassium oxide (K_2_O), 160; calcium (Ca), 91; magnesium (Mg), 9; sodium (Na), 1; sulfur (S), 5; iron (Fe), 4; manganese (Mn), 0.2; copper (Cu), 0.1; boron (B), 2; and zinc (Zn), 0.1; electrical conductivity (EC) was 0.5–0.7 mS cm^−1^. Mustard seeds (CN Seeds, Ltd., UK; 1 g) were sown per container, which represented one replicate. Four containers were used under each lighting condition. The containers were arranged randomly and systematically rotated every day to improve the uniformity of light exposure. Plants were watered daily with a light mist of tap water. Experiments were performed in closed controlled-environment growth chambers. Day/night temperatures of 21°C/17 ± 2°C were established with a relative air humidity of 50–60%.

### Lighting Conditions

**Figure 1:**
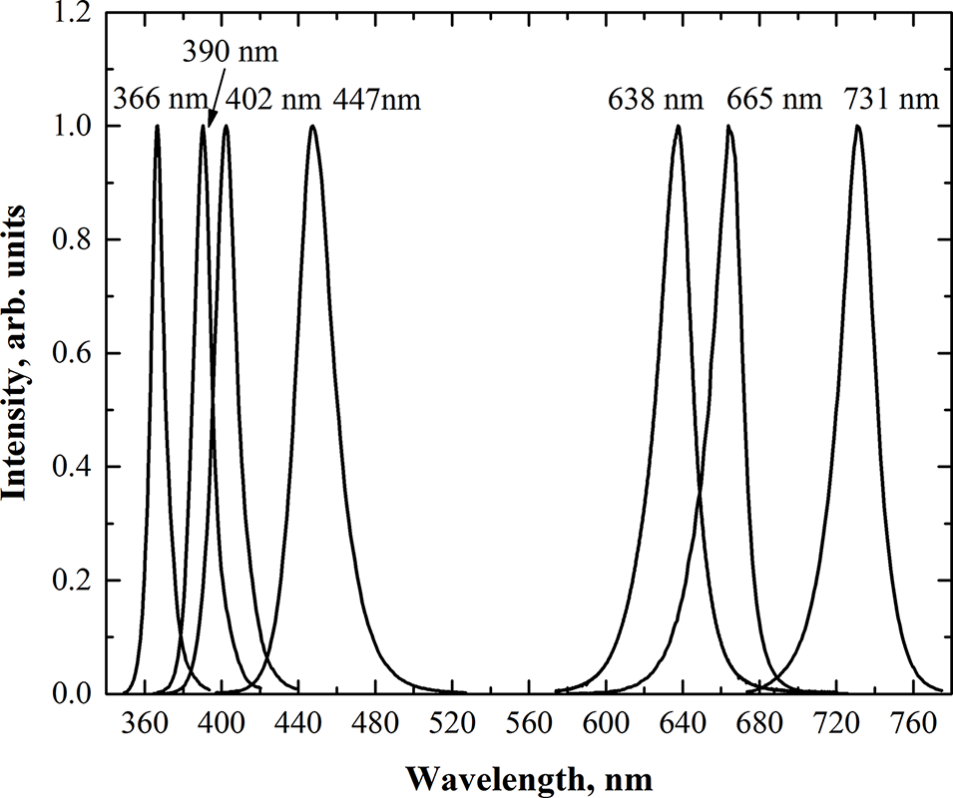
Relative spectral distribution of the LEDs used in the lighting equipment.

Microgreens were cultivated under custom-made lighting equipment containing five separate modules. Each module contained four main groups of high-power LEDs with different wavelengths. The main photosynthetic photon flux was provided by blue, red, deep-red, and far-red LEDs with peak wavelengths of 447 nm (Luxeon LXHL-LR3C; Philips Lumileds Lighting Co., USA), 638 nm (Luxeon LXHL-LD3C; Philips Lumileds Lighting Co., USA), 665 nm (Luxeon Rebel LXM3-PD01-0300; Philips Lumileds Lighting Co., USA), and 731 nm (L735-05-AU, Epitex Inc., Japan), respectively. Three modules were equipped with the fifth group of supplemental high-power UV-A LED-emitting wavelengths of 366 nm (NCSU033B, Nichia Corp., Japan), 390 nm (NCSU034B, Nichia Corp., Japan), and 402 nm (ACULED VHL ACL01–SC–UUUU–E05-C01-L-U000, PerkinElmer, Inc., USA). The emission spectra of LEDs were measured using a photonic multichannel analyzer (Hamamatsu PMA-12, Japan) in the laboratory before mounting them on lighting fixtures. The spectra of each LEDs group were rescaled (normalized) to the relative intensity of value 1 at the peak of wavelength (**Figure 1**). According to standards [e.g., ISO-21348:2007 (International Organization for Standardization, 2007)], the light with wavelengths below 400 nm is defined as UV. However, in human vision science, colorimetry, and photometry, radiation ranging from 380 to 400 nm is defined as visible. On the other hand, in semiconductor optoelectronics and lighting technologies, the UV radiation range is extended to slightly higher wavelengths (405 or 410 nm). Consequently, there is no strict boundary between UV and visible light in science and technology. Therefore, LED with a peak wavelength of 402 nm was considered as UV-A LED in this study.

All LED groups were controlled independently by custom-made high-power current regulators with digital controls. The lighting regime was pre-programmed by a controller unit with the help of a remote computer. Two different experiments with replications were carried out to determine the effects of UV-A on microgreens. **Table 1** presents the lighting regimes used in both experiments. In the first experiment (EXP1), the duration of UV-A irradiance was 10 h (from 8:00 a.m. to 6:00 p.m.). In the second experiment (EXP2), the duration of UV-A was 16 h (from 6:00 a.m. to 10:00 p.m.), simultaneously with the blue, red, deep-red, and far-red LEDs. Each lighting module reached a total photon flux density (TPFD) of 300 μmol m^−2^ s^−1^, as determined by a photometer-radiometer RF-100 (Sonopan, Poland). The photosynthetic photon flux density (PPFD) was 298 μmol m^−2^ s^−1^. The irradiance and TPFD of UV LEDs were measured using a UV-enhanced calibrated silicon photodiode and Labsphere illumia^®^Pro LEDs characterization system. The surface area under the lighting unit was approximately 0.22 m^2^, which is sufficient for the simultaneous growth of plants in amounts sufficient for the acquisition of statistically reliable data.

**Table 1:** Total (PFD) and photosynthetic (PPFD) photon flux densities (μmol m^–^^2^ s^–^^1^) and UV-A irradiance (W m^–^^2^), at the crop level produced by LEDs with different peak emission wavelengths.

Experiments	Supplemental UV-A radiation PFD, μmol m^–2^ s^–1^/W m^–2^				Standard irradiance* PFD, μmol m^–2^ s^–1^					
					PPFD					
	Photoperiod of UV-A (from–to, h)	UV-A 366 nm	UV-A 390 nm	UV-A 402 nm	B 447 nm	R 638 nm	DR 665 nm	FR 731 nm	Total PPFD	Total PFD
EXP 1	10 h (8 a.m.–6 p.m.)	12.4/4.0	12.4/3.8	12.4/3.7	21	122	155	2.2	298	300
EXP 2	16 h (6 a.m.–22 p.m.)									

*Photoperiod of standard irradiance—16 h. EXP, experiment; UV-A, ultraviolet A; B, blue; R, red; DR, deep-red; FR, far-red; PPFD, photosynthetic photon flux density (400–700 nm); PFD, photon flux density of plant photomorphogenetic response wavelength range (300–800 nm).

### Sampling and Destructive Measurements

Microgreen cotyledons, with stems, were harvested near the ground level. Samples were harvested from the center of the container, leaving plants in the 1.5-cm edge as a guard. Twenty plants were randomly selected from each replicate and used for biometric measurements and the determination of fresh weight to dry weight ratio and chlorophyll index. Three biological replicates were harvested for each biochemical analyses.

The leaf area of microgreens was measured using the WinDIAS meter (Delta-T Devices Ltd., UK). The dry and fresh weight of microgreens was determined by the gravimetric method using an electronic analytical balance (Mettler Toledo AG64, Columbus, OH, USA). Plants were dried in an oven at 105°C for 24 h (VENTICELL 222, MBT, Czech Republic) until a constant weight was achieved.

### Determination of Chlorophyll Content

The chlorophyll index was determined using a DUALEX SCIENTIFIC^™^ meter (Force-A, France), based on light transmission measurements in the red light spectrum.

### Phytochemical Analysis

#### Determination of Total Phenolic Content (TPC)

The TPC was determined spectrophotometrically, as described previously (Ragaee et al., 2006). Fresh plant tissue (1 g) was weighed on an analytical balance (Mettler Toledo AG 64, USA), placed in a 15-ml polypropylene centrifuge tube (VWR International, USA) and immediately frozen in 10 ml of liquid nitrogen (N_2_). Then, 10 ml of 80% methanol was added to the sample. The extract was shaken for 30 min in an orbital shaker (OS-20, Biosan Ltd., Latvia) and then centrifuged for 20 min at 2,012 × *g* (Hermle Z300K, Germany). The supernatant was filtered through a 70-mm Whatman^®^ Grade No. 1 qualitative filter paper. Then, 1 ml of the filtrate was diluted with 1 ml Folin–Ciocalteu reagent (1:10) and 2 ml of 7.5% sodium carbonate (Na_2_CO_3_) solution. After 20 min, the absorbance of the mixture was measured using a spectrophotometer (M501, Spectronic Camspec Ltd., UK) at 765 nm. The TPC in fresh plant tissues was calculated using a standard curve of gallic acid (*R* ^2^ ≥ 0.95). Data are presented as TPC per gram dry weight of microgreens.

#### Determination of Total Anthocyanin (TA) Content

To extract anthocyanins, fresh plant tissue (300 mg) was weighed on an analytical balance (Mettler Toledo AG 64, USA), placed in a 15-ml polypropylene centrifuge tube (VWR International, USA), and immediately frozen in 3 ml of liquid N_2_. Then, 5 ml of 2% HCl-methanol solution was added to the sample. The extract was shaken for 48 h in an orbital shaker (OS-20, Biosan Ltd., Latvia) and then centrifuged for 15 min at 1,446 × *g* (Hermle Z300K, Germany). The supernatant was filtered through a 70 mm Whatman^®^ Grade No. 1 qualitative filter paper. The TA content was determined spectrophotometrically, as described previously (Stanciu et al., 2009). The pH-differential method is based on colored oxonium predomination (0.025 M potassium chloride [KCl] buffer; pH 1) *vs*. a colorless hemiketal (0.4 M sodium acetate [CH_3_COONa] buffer; pH 4.5). The dilution factor of the extract and buffer was 6, and sample absorption values were measured using a spectrophotometer (M501, Spectronic Camspec Ltd., UK) at 420, 520, and 700 nm. The TA content was expressed as cyanidin 3-glucoside equivalents using a molar extinction coefficient of 25.74 M^−1^ cm^−1^ and a molecular weight of 485 g mol^−1^. Data are presented as TA per gram dry weight of microgreens.

#### Determination of DPPH Free-Radical Scavenging Activity

The radical scavenging activity of 2,2-diphenyl-1-picrylhydrazyl (DPPH) was evaluated as described previously (Ragaee et al., 2006), with slight modifications. Methanol extracts used for the total phenolic content assay were diluted with the DPPH solution. Absorbance was measured after 16 min using a spectrophotometer (M501, Spectronic Camspec Ltd., UK) at 515 nm. Data are presented as DPPH free-radical scavenging activity per gram dry weight of microgreens.

#### Determination of Ascorbic Acid (AA) Content

The AA content was assessed using a spectrophotometric method (Janghel et al., 2007), based on the ability of the ascorbate ion to reduce methyl viologen to a stable blue-colored free-radical ion. Samples were prepared from 1 g of fresh plant material that was homogenized with 10 ml of 5% metaphosphoric acid and centrifuged at 2,012 × *g* for 5 min. Then, 2 ml each of methyl viologen and 2 M NaOH were mixed with 1 ml of sample extraction. After 2 min, absorption was measured using a spectrophotometer (M501, Spectronic Camspec, Ltd., U.K) at a wavelength of 600 nm. The concentration of AA in fresh plant tissues was determined using the calibration data of AA standards. Data are presented as AA content per gram dry weight of microgreens.

#### Determination of **α**-Tocopherol Content

The α-tocopherol content was evaluated using high-performance liquid chromatography (HPLC) on a Pinnacle II Silica column (Restek, USA; 5-μm particle size; 150 mm × 4.6 mm), as described previously (Fernandez-Orozco et al., 2003). Tocopherols were extracted from fresh plant tissues using pure hexane (1:10) by centrifugation at 349 × *g* for 5 min. The supernatant was filtered through a 0.45-μm polytetrafluoroethylene (PTFE) membrane syringe filter (VWR International, USA). The HPLC 10A system, equipped with an RF-10A fluorescence detector (Shimadzu, Japan), was used for analysis. Peaks were detected at an excitation wavelength of 295 nm and an emission wavelength of 330 nm. The mobile phase (0.5% isopropanol in hexane) was used at a flow rate of 1 ml min^−1^. The α-tocopherol was identified according to the analytical standard. The α-tocopherol content is expressed per gram dry weight of microgreens.

#### Determination of Carotenoid Content

The contents of lutein-zeaxanthin and β-carotene were evaluated using HPLC on a YMC carotenoid column (YMC, Japan; 3-µm particle size; 150 mm × 4.0 mm), as described previously (Edelenbos et al., 2001). Plant tissue (1 g) was ground in liquid N_2_. Then, 10 ml of 80% acetone was added to the sample and mixed. The sample was centrifuged at 5,000 × *g* for 15 min. The supernatant was filtered through a 0.22-µm nylon membrane syringe filter (VWR International, USA). The HPLC 10A system (Shimadzu, Japan), equipped with a diode array (SPD-M 10A VP) detector, was used for the analysis. Peaks were detected at 440 nm. The mobile phase consisted of solvent A (80% methanol and 20% water) and solvent B (100% ethyl acetate). Carotenoids were identified according to the standards. Carotenoid contents are expressed per gram dry weight of microgreens by the calculation of the ratio of fresh weight to dry weight.

#### Determination of Macro- and Microelement Content

The contents of macro- and microelements in microgreens were determined using the microwave digestion technique, in combination with inductively coupled plasma optical emission spectrometry (ICP-OES) (Araújo et al., 2002; Barbosa et al., 2015). Dried microgreens (0.5 g) were completely digested with 65% nitric acid (HNO_3_) and 30% hydrogen peroxide (H_2_O_2_) in a 5:3 ratio using the microwave digestion system Multiwave GO (Anton Paar GmbH, Austria). To digest the sample, the temperature was increased to 150°C over 3 min, and the sample was digested for 10 min. Then, the temperature was increased to 180°C over 10 min, and the sample was digested for 10 min. The mineralized samples were diluted with deionized water to a final volume of 50 ml. The elemental profile was analyzed using an ICP optical emission spectrometer (SPECTRO Genesis, SPECTRO Analytical Instruments, Germany) using the following operating conditions: 1,300 W RF power, 12 L min^−1^ plasma flow, 1 L min^−1^ auxiliary flow, 0.8 L min^−1^ nebulizer flow, and 1 ml min^−1^ sample uptake rate. Different analytical wavelengths were chosen for different elements: B (I), 249.773 nm; Ca (II), 445.478 nm; Cu (I), 324.754 nm; Fe (II), 259.941 nm; potassium (K [I]), 766.491 nm; Mg (II), 279.079 nm; Mn (II), 259.373 nm; Na (I), 589.592 nm; phosphorous (P [I]), 213.618 nm; S (I), 182.034 nm; and Zn (I), 213.856 nm. The calibration standards were prepared by diluting a stock multi-element standard solution (1,000 mg L^−1^) in 6.5% (v/v) HNO_3_ and by diluting stock P and S standard solutions (1,000 mg L^−1^) in deionized water. The calibration curves for all studied elements were in the range of 0.01–400 mg L^−1^. The contents of macro and microelements are expressed per gram dry weight of microgreens.

#### Determination of Nitrate Content

Nitrate concentration in microgreens was measured using the potentiometric method (Geniatakis et al., 2003) with an ion meter (Oakton, USA) and combined nitrate ion selective electrode HI4113 (HANNA instruments, USA). The ionic strength adjustor (ISA) contained 0.02 mol L^−1^ aluminum sulfate (Al_2_[SO_4_]_3_) (Poch, Poland), 0.01 mol L^−1^ silver sulfate (Ag_2_SO_4_) (DeltaChem, Czech Republic), and 0.02 mol L^−1^ boric acid (H_3_BO_3_) (Poch, Poland). Dry sample (0.2 g) was added to 20 ml of water: ISA solution (1:1, v/v) and extracted in an ultrasound bath for 10 min. All measurements were performed after the sensor signal had been stabilized for 3 min. The results are presented as nitrate content in fresh plant tissues.

### Statistical Analysis

Statistical analysis was performed using the XLSTAT statistical and data analysis solution for Microsoft Excel (Addinsoft, Paris, France). Data are presented as mean ± standard deviation (SD). Differences between means were evaluated for each experiment using the Duncan’s Multiple Range test at a significance level of *P* ≤ 0.05. Principal component analysis (PCA) was performed at the significance level of *P* ≤ 0.05.

## Results

**Figure 2:**
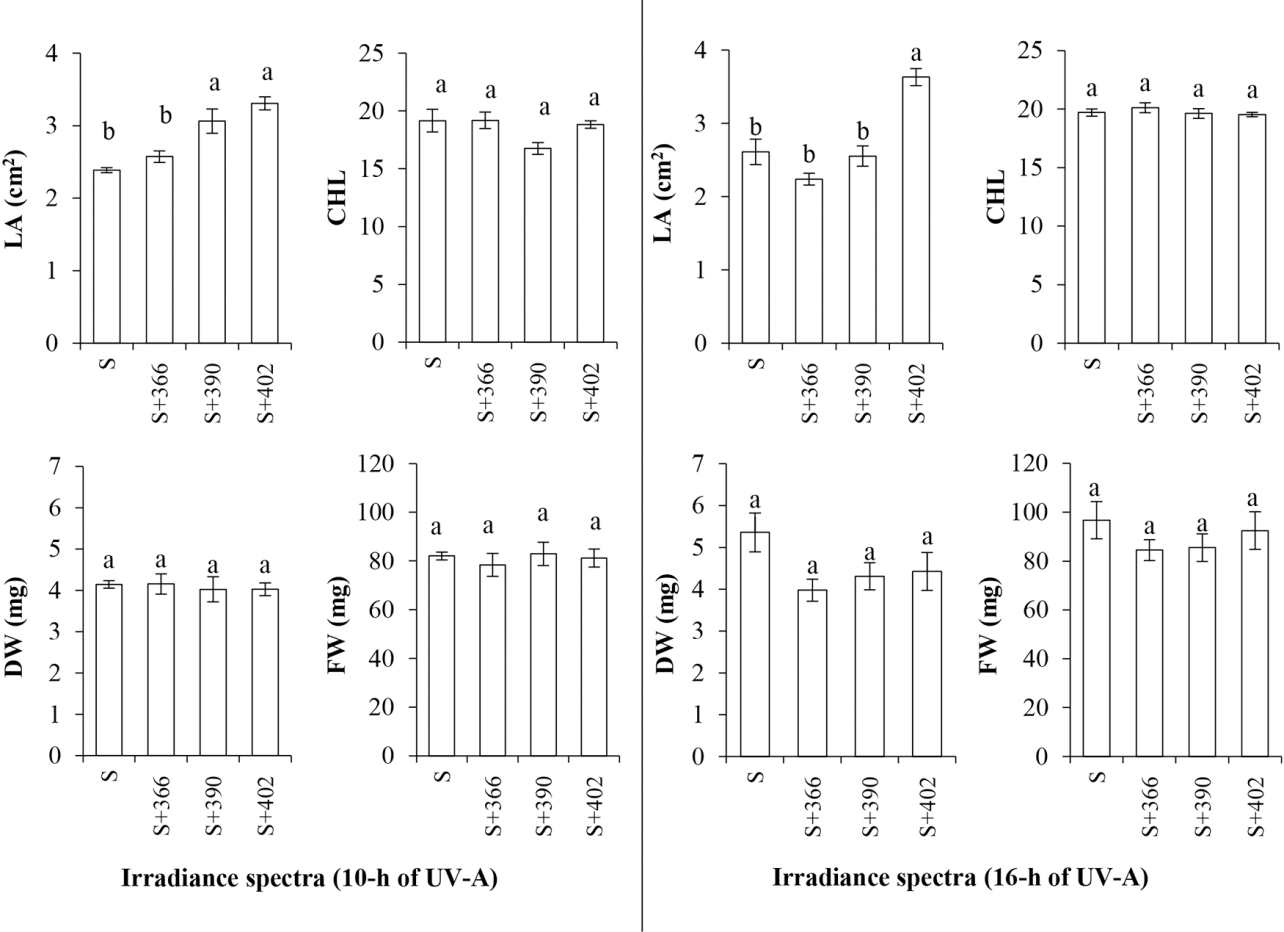
The effect of supplemental UV-A LED radiation on leaf area (LA), chlorophyll index (CHL), dry weight (DW), and fresh weight (FW), of mustard microgreens. S, standard irradiance. Different letter(s) indicates a significant difference at P ≤ 0.05 by the Duncan’s Multiple Range test.

Supplemental UV-A radiation at different wavelengths and durations had variable effects on the growth characteristics of mustard microgreens (**Figure 2**). Increasing the wavelength of supplemental UV-A radiation (from 366 to 402 nm) at 10 h under standard illumination significantly increased the leaf area of microgreens. However, only 402 nm at 16 h irradiance had a positive effect on the increase in leaf area. Neither the supplemental UV-A wavelengths under standard illumination nor the UV-A irradiance duration had a significant effect on the fresh and dry weight of microgreens or on the chlorophyll index (**Figure 2**).

**Figure 3:**
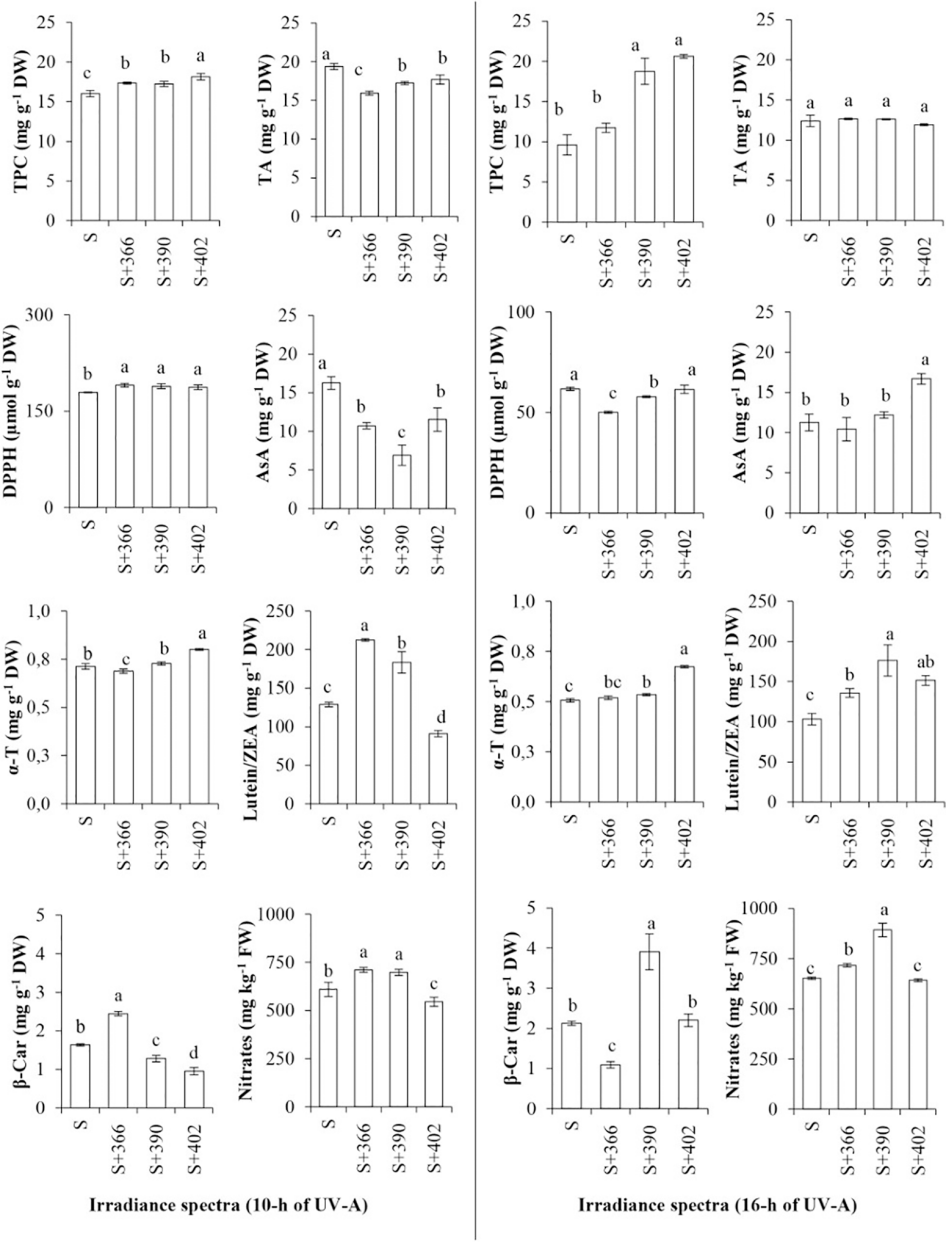
The effect of supplemental UV-A LED radiation on nutritional quality characteristics of mustard microgreens. TP, total phenols; TA, total anthocyanins; DPPH, DPPH• radical-scavenging activity; AA, ascorbic acids; α-T, α-tocopherol; L/Z, lutein/zeaxanthin; BC, β-carotene; NO_3_, nitrates; S, standard irradiance. Different letter(s) indicates a significant difference at P ≤ 0.05 by the Duncan’s Multiple Range test.

Additionally, supplemental UV-A irradiance significantly affected the nutritional quality of mustard microgreens (**Figure 3**). Our data revealed that most of the supplemental UV-A wavelengths resulted in a significant increase in the TPC of microgreens at both irradiance durations. DPPH free-radical scavenging activity was higher at the 10-h duration of UV-A irradiance (EXP1), whereas TA and AA contents decreased under these lighting conditions. Longer duration of UV-A irradiance (EXP2) had no effect on the TA content, whereas 366 and 390 nm of radiation decreased DPPH free-radical scavenging activity. The content of AA increased significantly after 16-h exposure to 402 nm UV-A, while the α-tocopherol content was significantly higher at 402 nm, regardless of the duration, and at 390 nm and 16-h irradiance compared with other treatments. Supplemental UV-A wavelengths at both irradiance durations caused an increase in lutein/zeaxanthin content, except 402 nm at 10 h irradiance. The content of β-carotene was significantly higher after exposure to 366 nm UV-A LED for 10 h and 390 nm UV-A LED for 16 h. Nitrate content significantly increased upon exposure to 366- and 390-nm wavelengths, independent of the duration of UV-A radiation, while a decrease in nitrate content was observed in response to 402-nm wavelength for 10 h. Different UV-A wavelengths showed no effect on nitrate content at 16-h exposure.

The effect of supplemental UV-A irradiance on mineral element content was wavelength- and duration-dependent (**Table 2**, **Table 3**). Shorter durations (10 h; EXP1) of all wavelengths negatively affected the content of the macroelements including P, Ca, Na, and Mg. A similar decrease was observed in all measured microelements. The content of S increased under UV-A wavelengths (**Table 2**). Longer duration (16 h) of 390-nm wavelength positively affected P, Ca, and B contents, while 16 h at 402-nm wavelength increased P, Mg, Mn, Zn, and Cu contents.

**Table 2:** The effect of supplemental UV-A LED radiation on the macro elements of mustard microgreens.

Variants	Macroelements, mg g^−1^ DW					
	P	K	Ca	Mg	Na	S
UV-A 10 h (EXP 1)						
UV-A 16 h (EXP 2)						
S	8.62±0.08a	26.4±0.16a	16.4±1.55a	1.16±0.002a	3.79±0.04a	10.4±0.48c
S+366 nm	7.56±0.19c	25.4±0.22b	13.2±1.07b	1.12±0.002d	3.34±0.05c	11.2±0.16b
S+390 nm	7.95±0.10b	26.5±0.10a	13.9±0.70ab	1.14±0.002b	3.64±0.01b	13.7±0.08a
S+402 nm	7.46±0.02c	25.0±0.17b	12.4±1.08b	1.13±0.002c	3.27±0.03c	14.0±0.32a
S	6.88±0.05c	20.6±0.10c	20.7±0.98ab	1.15±0.001b	4.73±0.01b	13.4±0.19c
S+366 nm	6.70±0.05c	22.0±0.06b	20.9±0.34ab	1.13±0.001c	4.68±0.01c	13.3±0.10c
S+390 nm	7.22±0.13b	23.2±0.21a	23.0±1.67a	1.13±0.002c	4.68±0.01c	15.0±0.17b
S+402 nm	7.41±0.05a	20.4±0.18c	20.2±0.70b	1.16±0.001a	4.78±0.01a	16.0±0.14a

S, standard irradiance; DW, dry weight; P, phosphorus; K, potassium; Ca, calcium; Mg, magnesium; Na, sodium; S, sulfur. Different letter(s) within the column indicates a significant difference at P ≤ 0.05 by the Duncan’s Multiple Range test.

**Table 3:** The effect of supplemental UV-A LED radiation on the microelements of microgreens.

Variants	Microelements, mg 100 g^−1^ DW				
	Mn	Fe	Zn	Cu	B
UV-A 10 h (EXP 1)					
UV-A 16 h (EXP 2)					
S	7.01±0.08a	16.5±0.24a	5.90±0.05a	0.59±0.005a	1.96±0.05a
S+366 nm	5.80±0.10c	14.6±0.09c	5.00±0.04c	0.47±0.008b	1.60±0.02c
S+390 nm	6.43±0.05b	15.2±0.09b	5.25±0.02b	0.47±0.005b	1.80±0.02b
S+402 nm	5.90±0.06c	12.8±0.06d	4.92±0.04c	0.45±0.005c	1.60±0.02c
S	8.71±0.04c	11.5±0.04a	4.20±0.03c	0.42±0.008b	1.91±0.02b
S+366 nm	8.73±0.04c	10.4±0.05c	4.04±0.01d	0.40±0.005c	1.94±0.03b
S+390 nm	8.95±0.05b	10.3±0.08d	4.33±0.04b	0.43±0.008b	2.03±0.03a
S+402 nm	9.98±0.03a	11.2±0.02b	4.66±0.01a	0.47±0.001a	1.97±0.01ab

S, standard irradiance; DW, fresh weight; Mn, manganese; Fe, iron; Zn, zinc; Cu, copper; B, boron. Different letter(s) within the column indicates a significant difference at P ≤ 0.05 by the Duncan’s Multiple Range test.

**Figure 4:**
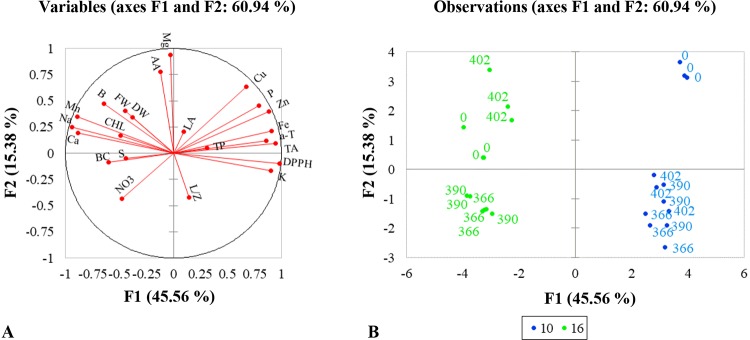
Multivariate principal component analysis showing the effect of supplemental UV-A LED radiation on mustard microgreens. **(A)** Correlation circle, summarizing metabolic relations between investigated parameters under different lighting conditions. LA, leaf area; FW, fresh weight; DW, dry weight; CHL, chlorophyll index; TP, total phenols; TA, total anthocyanins; DPPH, DPPH• radical-scavenging activity; AA, ascorbic acids; α-T, α-tocopherol; L/Z, lutein/zeaxanthin; BC, β-carotene; NO_3_, nitrates; P, phosphorus; K, potassium; Ca, calcium; Mg, magnesium; Na, sodium; S, sulfur; Mn, manganese; Fe, iron; Zn, zinc; Cu, copper; B, boron. **(B)** PCA scatter plot, indicating distinct growth and metabolism in mustard under different lighting conditions. 0—standard irradiance without UV-A radiation; 366, 390, 402—wavelengths (nm) of UV-A radiation; 10, 16—duration in hours of UV-A radiation in standard irradiance. The significance level was set at P ≤ 0.05 for the model.

To compare the responses of mustard microgreens to different wavelengths and durations of UV-A radiation, we performed PCA (**Table 4**). The first five principal components (F1–F5) were associated with eigenvalues of more than 1 and accounted for approximately 89.68% of the cumulative variance (**Table 4**). F1, which explained 45.56% of the variance, was mainly attributed due to the DPPH radical scavenging activity, TA, α-tocopherol, and mineral elements such as K, P, Fe, Cu, and Zn. Other minerals elements like Ca, Na, Mn, and B contributed negatively to F1. F2 accounted for 15.38% of the total variance. Factors that contributed to F2 include AA and Mg. F3 explained 11.74% of the variance; this included factors such as lutein/zeaxanthin, β-carotene, and nitrates. F4 accounted for 10.48% of the total variation in the population and was mainly ascribed to leaf area, TPC, and S content. F5 accounted for 6.53% of the total variation and was mainly attributed to the fresh and dry weight of mustard microgreens. **Figure 4** illustrates the PCA of the first two components (F1 and F2). The correlation circle and matrix (**Figure 4A**, **Supplemental Table S1**) illustrate the relationships among the different variables (i.e., growth and nutritional quality components), where two vectors with an angle less than 90° are positively correlated, and two vectors with an angle higher than 90° are negatively correlated. For example, the DPPH free-radical scavenging activity and TA content were strongly positively correlated with each other. Similarly, α-tocopherol content showed a positive correlation with the contents of mineral elements such as K, Fe, and Zn. These parameters (DPPH•, TA, and α-tocopherol) were negatively correlated with Ca, Na, and α-tocopherol contents. Ca was strongly positively correlated with Na, Mn, B, while P showed a strong positive correlation with Fe, Cu, and Zn. The F1 and F2 score plots (**Figure 4B**) categorized treatments into four groups. The lower right quadrant included the effect of all supplemental UV-A wavelengths at shorter irradiance duration (10 h), which differed from the treatment without supplemental UV-A irradiance. The lower negative side of F1 included the effect of 366- and 390-nm UV-A wavelengths at a longer duration (16 h), and the upper left quadrant showed a different effect of the 402-nm UV-A LED.

**Table 4:** Eigenvalue, factor scores, and contribution of the first five principal component axes to variation in mustard microgreens under supplemental UV-A LED radiation.

Parameter	F1	F2	F3	F4	F5
LA	0,030	0,108	-0,307	0,508	0,110
CHL	−0,152	0,091	0,039	−0,214	−0,169
DW	−0,117	0,184	−0,210	−0,127	0,607
FW	−0,138	0,214	−0,228	−0,023	0,545
DPPH	0,304	−0,051	−0,033	0,001	0,100
TP	0,096	0,028	0,174	0,547	−0,022
AA	−0,038	0,414	0,095	0,096	−0,174
TA	0,292	0,050	0,025	−0,097	−0,056
α-T	0,266	0,063	−0,139	0,256	−0,051
L/Z	0,044	−0,225	0,397	0,167	0,192
BC	−0,136	−0,027	0,374	0,153	0,312
NO_3_	−0,147	−0,230	0,415	0,061	0,217
K	0,279	−0,088	0,151	−0,055	0,095
Ca	−0,272	0,102	0,198	−0,046	−0,084
Mg	−0,009	0,500	0,015	0,002	−0,082
Na	−0,290	0,133	0,080	−0,006	−0,055
P	0,244	0,241	0,217	0,046	0,067
S	−0,185	−0,046	−0,132	0,454	−0,083
Mn	−0,276	0,185	0,076	0,105	−0,098
Fe	0,281	0,114	0,099	−0,114	0,129
Cu	0,209	0,339	0,211	−0,042	0,003
Zn	0,272	0,212	0,139	0,025	0,052
B	−0,199	0,252	0,256	−0,023	−0,013
Eigen value	10,48	3,54	2,70	2,41	1,50
Variability (%)	45,56	15,38	11,74	10,48	6,53
Cumulative %	45,56	60,94	72,68	83,16	89,68

LA, leaf area; FW, fresh weight; DW, dry weight; CHL, chlorophyll index; TP, total phenols; TA, total anthocyanins; DPPH, DPPH• radical-scavenging activity; AA, ascorbic acids; α-T, α-tocopherol; L/Z, lutein/zeaxanthin; BC, β-carotene; NO_3_, nitrates; P, phosphorus; K, potassium; Ca, calcium; Mg, magnesium; Na, sodium; S, sulfur; Mn, manganese; Fe, iron; Zn, zinc; Cu, copper; B, boron.

## Discussion

Light affects plants *via* specific photoreceptors, which absorb light at different wavelengths. For example, phytochromes A–E (PHYA–E) absorb red/far-red light, while cryptochromes 1 and 2 (CRY1 and CRY2), phototropins 1 and 2 (PHOT1 and PHOT2), and Zeitlupe (ZTL) absorb blue and UV-A light. The UV resistance locus 8 (UVR8) is the only photoreceptor identified to date that is triggered by UV-B light. Photoreceptors initiate signaling pathways that lead to the expression of light-responsive genes that regulate growth, development, and metabolite biosynthesis (Huché-Thélier et al., 2016; Verdaguer et al., 2017; Neugart and Schreiner, 2018). Published reports on UV-A mediated responses present contradictory results, which might be due to other light wavelengths used together with those of UV-A (Verdaguer et al., 2017). For example, while UV-A did not have significant effects on pea (*Pisum sativum* L., cv. “Shen Chun”) seedlings (Liu and Yang, 2012; Wenke and Qichang, 2012) and eggplant (*Solanum melongena* L. cv. “Black beauty”) (Dáder et al., 2014), it had negative effects on the growth of pepper (*Capsicum annuum* L. cv. “California Wonder”) (Dáder et al., 2014) and different cultivars of lettuce (*Lactuca sativa* L.) (Krizek et al., 1998; Tsormpatsidis et al., 2008). Positive effects of UV-A have been reported on radish (*Raphanus sativus* L.) (Tezuka et al., 1994). However, the above-mentioned treatments were carried out using different kinds of cutoff filters or supplemental photosynthetically active radiation (PAR) and UV lamps, with a wide range of UV-A radiation. The rapid development of UV-emitting diodes makes it possible to add single UV-A wavelengths to the light spectrum in CEA lighting, which enables a more accurate assessment of the effect of those single wavelengths. Only few studies have been published on the effects of UV-A on vegetables, and these studies show stimulatory effects of UV-A on the growth of some vegetables (Brazaitytė et al., 2010; Chang and Chang, 2014; Khoshimkhujaev et al., 2014; Brazaitytė et al., 2015; Mickens et al., 2019) but inhibitory (Brazaitytė et al., 2009; Vaštakaitė et al., 2015; Jensen et al., 2018) or no effects on others (Li and Kubota, 2009). Our investigations with a standard visible LED lighting system revealed a positive effect of 390-nm UV-A (for 10 h) and 402-nm UV-A (for 10 and 16 h) on the leaf area of mustard microgreens (**Figure 2**). However, none of the supplemented UV-A wavelengths had a significant effect on biomass. These results suggest that the supplementation of blue, red, far-red (visible) LEDs with different UV-A wavelengths has no negative impact on the biomass of mustard microgreens.

Our study revealed no effect of UV-A irradiance on the chlorophyll content of mustard (**Figure 2**). These results are consistent with those of other studies on various vegetables, such as lettuce, tomato, cucumber (*Cucumis sativus*), and pepper (Caldwell and Britz, 2006; Brazaitytė et al., 2009; Brazaitytė et al., 2010; Li and Kubota, 2009; Chang and Chang, 2014; Dáder et al., 2014). Red pak choi (*Brassica rapa* var. *Chinensis*, “Rubi F_1_”) plants grown under LED-based artificial sunlight research module (ASRM) lamps showed significantly lower chlorophyll content than plants grown under white LED treatments without UV-A (Mickens et al., 2019) as well as eggplant under UV-A irradiance (Dáder et al., 2014). Pea seedlings exposed to supplemental UV-A fluorescent lamps showed lower chlorophyll b content than plants without UV-A treatment (Wenke and Qichang, 2012). The chlorophyll content is one of the many indices that reflect the performance of the photosynthetic system. Photoreceptors, especially PHYs, are important for the photoprotection of the photosynthetic apparatus from UV-A. Kreslavski et al. (2016) suggested that PHYs affect the resistance of the photosynthetic apparatus to UV-A by changing pigment composition and inducing the transcription of some antioxidant genes, such as *APX1* and *tAPX*. Pre-illumination with red light of indoor-grown lettuce and *Arabidopsis thaliana* seedlings enhanced their resistant to UV-A through the stimulation of antioxidant enzymes and UV-A-absorbing pigments and a reduced loss of carotenoids and chlorophylls. These effects are mediated by PHYB as revealed by experiments with the PHYB mutant *hy3* of *A. thaliana (LIT)* (Kreslavski et al., 2016; Verdaguer et al., 2017). In our study, UV-A exposure occurred under conditions where the spectrum contained high proportions of red light. It could be presumed that the higher amount of red light increased resistance to UV-A irradiance in mustard microgreens, which would explain the lack of chlorophyll loss. Verdaguer et al. (2017) concluded that UV-A can cause photodamage to photosystem II (PSII), but such damage can be reduced or even annulled by mechanisms that weaken the effect UV-A radiation such as the accumulation of compounds that absorb UV-A wavelengths.

Carotenoids, such as xanthophylls, are light-harvesting pigments in chloroplasts that are involved in the protection of plants against photooxidative stress, and their synthesis is controlled by blue/UV-A/UV-B photoreceptors (Jahns and Holzwarth, 2012; Verdaguer et al., 2017; Neugart and Schreiner, 2018). Our experiments confirmed the protective function of xanthophylls, such as lutein/zeaxanthin, and showed their increase in mustard microgreens under most supplemental UV-A wavelengths at both irradiance durations. However, irradiance with 402-nm UV-A for 10 h resulted in a reduction in lutein/zeaxanthin content (**Figure 3**). Lefsrud et al. (2008) reported that UV-A (400 nm) LEDs have no positive effect on the accumulation of lutein. Therefore, it could be inferred that the protective function of xanthophylls is expressed more at shorter supplemental UV-A wavelengths in the blue, red, and far-red illumination spectrum. Higher lutein content also was determined in broccoli (*Brassica oleracea*) sprouts (Moreira-Rodríguez et al., 2017). However, supplemental UV-A LEDs added to fluorescence lamp illumination had no effect on xanthophylls in red baby leaf lettuce (Li and Kubota, 2009) and lutein in most varieties of green and red lettuce in the greenhouse (Caldwell and Britz, 2006). Additionally, UV-A treatments showed no effect on β-carotene content in green and red lettuce and baby leaf lettuce (Caldwell and Britz, 2006; Li and Kubota, 2009; Samuolienė et al., 2013). Our experiments determined that the content of β-carotene in mustard microgreens increased when grown under 366-nm UV-A at 10-h irradiance and under 390-nm UV-A at 16-h irradiance.

One of the more heavily researched responses of plants to UV radiation is the induction and synthesis of flavonoids and related phenolic compounds. The accumulation of such UV-absorbing compounds in epidermal tissue results in the reduction of UV transmittance and plays an important role in plant acclimation to UV radiation in the environment, including UV-A (Tsormpatsidis et al., 2008; Verdaguer et al., 2017; Neugart and Schreiner, 2018). UV-A irradiance stimulates the expression of UV-protective genes and the accumulation of phenolics. UV-A induces the expression of genes involved in the flavonoid biosynthesis pathway, such as phenylalanine ammonia-lyase (*PAL*), chalcone synthase (*CHS*), production of anthocyanin pigment 1 (*PAP1*), and dihydroflavonol 4-reductase (*DFR*) (Morales et al., 2013; Verdaguer et al., 2017; Alrifai et al., 2019). However, the different effects of UV-A on the phenolic content of various plants, including vegetables, suggest different roles of the UV-A/blue light photoreceptors during UV-A-induced metabolite biosynthesis (Verdaguer et al., 2017). Previous studies indicate that UV-A exposure *via* UV-A lamps and UV-selective films causes an increase in TA content, flavonoid content, and TPC in red leaf lettuce (Voipio and Autio, 1995; Tsormpatsidis et al., 2008), specific phenolics in broccoli sprouts (Moreira-Rodríguez et al., 2017), polyphenols in *Perilla frutescens* (Iwai et al., 2010), and total phenols in pepper plants (Dáder et al., 2014). However, UV-A exposure showed no significant effect on phenolic compounds in pea seedlings (Liu and Yang, 2012; Wenke and Qichang, 2012) and eggplant (Dáder et al., 2014). UV-A LEDs allow for the evaluation of the effects of individual UV wavelengths. UV-A (380 nm) LEDs used as supplemental light sources to blue/red/far-red LED illumination increased the TPC of green baby leaf lettuce cv. ‘Thumper’ but had no effect on the anthocyanin content (Samuolienė et al., 2013). UV-A (373 nm) LEDs used as supplemental light sources to fluorescent lamps caused an increase in anthocyanins, but not phenolic compounds, in baby leaf cv. ‘Red Cross’ (Li and Kubota, 2009). Supplemental UV-A LEDs of 325 nm, in addition to the red LED, was more effective in the accumulation of anthocyanins in red leaf lettuce cv. ‘Red Fire’ than 340-nm LEDs (Goto et al., 2016), while UV-A (366 nm) increased the TPC and TA content of beet (*Beta vulgaris*) and red pak choi microgreens (Brazaitytė et al., 2015). Our experiments revealed an increase in the TPC of mustard microgreens under all supplemental UV-A LED wavelengths (366, 390, and 402 nm) at both durations of exposure, although a more pronounced effect was evident under a longer wavelength (**Figure 3**). However, the total anthocyanin content of mustard microgreens decreased under shorter UV-A, although longer UV-A had no significant effect. Zhou et al. (2007)suggested that UV-A-dependent anthocyanin biosynthesis in swollen hypocotyls of turnip (*B. rapa*) is mediated by a UV-A-specific photoreceptor but not by blue or UV-B photoreceptors. Their study of UV-A (360 nm) LEDs without UV-B radiation showed induction of anthocyanin biosynthesis, whereas the longer wavelength UV-A (395 nm) and visible light free of UV did not induce anthocyanin biosynthesis (Zhou et al., 2007). The authors determined that the expression of anthocyanin biosynthesis genes, such as *CHS* and *F3H* (naringenin 3-dioxygenase/flavanone 3-hydroxylase), was regulated only by UV-A exposure. Another study similarly reported that UV-A irradiance at wavelengths of 330–360-nm induced *CHS* expression, while UV-A with a wavelength longer than 400 nm did not (Takeda et al., 1994). These data were obtained using a range of light wavelengths, not only UV-A irradiance, which was different from the above-described experiment in turnip (Zhou et al., 2007). In our experiment, UV-A LEDs were used supplementary to other light wavelengths, which either decreased or had no effect on the anthocyanin content of mustard microgreens (**Figure 3**). This suggests the existence of different anthocyanin biosynthesis pathways that are regulated not by blue/UV-A photoreceptors but by red/far-red light photoreceptors. Such a presumption confirms the experiments of Zhou et al. (2007), where the upper part of the turnip hypocotyls displayed far-red-dependent anthocyanin biosynthesis. In *Arabidopsis* and grape (*Vitis vinifera* L.), UV-A-dependent anthocyanin biosynthesis was induced by blue or white light as well as by UV-A (Zhou et al., 2007). Therefore, such varying results indicate the need for further investigation of UV-A exposure on plants.

Other secondary metabolites, such as α-tocopherol and AA, also perform a protective function against unfavorable light conditions. It is well known that α-tocopherol is the main vitamin E compound located in the chloroplast envelope, thylakoid membranes, and plastoglobuli. It neutralizes the photosynthetically derived reactive oxygen species (ROS) and protects the photosynthetic apparatus from oxidative stress and lipid peroxidation during environmental stresses, such as high light intensity and UV-B radiation (Munné-Bosch, 2005; Hasanuzzaman et al., 2014). However, limited information is available on the effect of UV-A on α-tocopherol content in vegetables. It has been shown that UV-A exposure does not affect the α-tocopherol content of fresh leaves of lettuce, garland chrysanthemum (*Glebionis coronaria*), and spinach (*Spinacia oleracea*) (Yun et al., 2003). However, UV-A exposure decreased the content of α-tocopherol in baby leaf lettuce and increased it in beet and red pak choi microgreens (Samuolienė et al., 2013; Brazaitytė et al., 2015). Our experiment revealed that the most pronounced effect of UV-A on mustard was at a wavelength of 402 nm (**Figure 3**). Since the level of ROS increases during environmental stress, the level of tocopherols could be related to a stress tolerance response. If stress-tolerant plants usually increase their tocopherol content, the most sensitive plants will show a loss of this compound, when the stress takes place over a longer period of time, and tocopherol degradation exceeds its synthesis (Munné-Bosch, 2005). Our results did not show an increase in the content of α-tocopherol in mustard microgreens grown under supplemental UV-A (366 and 390 nm), and that could have been because mustard microgreens are sensitive to UV-A exposure. Additionally, some studies show that *α*-tocopherol has an interdependent relationship with carotenoids, some phenolics, anthocyanins, and plant growth (Trebst, 2003; Munné-Bosch, 2005). Our data partly confirm this finding: α-tocopherol content showed a strong positive correlation with DPPH (0.844) and anthocyanins (0.765), a moderate correlation with TPC (0.580) and leaf area (0.520), and a weak negative correlation with β-carotene (−0.437) (**Figure 4A**, **Supplemental Table S1**).

AA is a non-enzymatic antioxidant that is important not only for scavenging ROS but also as a cofactor of violaxanthin de-epoxidase (VDE). VDE regenerates tocopherol from the tocopheroxyl radical, thus providing membrane protection, and is involved in the regulation of cell division and elongation. Light quantity and quality are key factors affecting the synthesis of AA (Das and Roychoudhury, 2014; Akram et al., 2017; Ntagkas et al., 2018). However, UV stress, which induces the production of ROS, does not always result in the enhancement of AA content (Mittler, 2002). Our experiments confirmed this observation and revealed that only 402-nm supplemental UV-A increased the content of AA in mustard microgreens (**Figure 3**). In general, the application of UV-A radiation has been shown to have an uneven effect on AA accumulation in leafy vegetables. For example, UV-A radiation had no effect on the AA content of baby leaf lettuce (Li and Kubota, 2009) and pea seedlings (Liu and Yang, 2012; Wenke and Qichang, 2012) increased its content in green and purple basil (*Ocimum basilicum*) (Vaštakaitė et al., 2015) and red pak choi and decreased its content in beets and basil-cultivated indoors (Brazaitytė et al., 2015).

Generally, all analyzed metabolites not only play important protective roles against environmental stresses in plants but also have a beneficial impact on human health by acting as antioxidants that reduce the risk of cancer, cardiovascular disease, and neurodegenerative diseases (Andréa et al., 2010; Rouphael et al., 2018). However, plants accumulate not only beneficial compounds but also antinutrients, such as nitrates (Bian et al., 2015; Rouphael et al., 2018). Generally, nitrates are necessary for normal plant growth and are nontoxic to humans below a certain threshold (acceptable daily intake [ADI] of 3.7 mg kg^–1^ body weight). Light has been known as one of the major factors affecting nitrate metabolism through PHY- and elongated hypocotyl 5 (HY5)/HY5 homolog (HYH)–dependent signaling pathways, energy-related AMP-activated protein kinase (AMPK), sucrose non-fermenting (SNF)–related protein kinase 1 (SnRK1), chloroplastic thioredoxins, and PII proteins. Therefore, appropriate illumination conditions are important to optimize the accumulation of nitrate (EFSA, 2008; Lillo, 2008; Lin et al., 2013; Bian et al., 2015). However, no information is available on the effect of UV-A on nitrate metabolism and the role of blue/UV-A photoreceptors in this process. Our experiments showed that supplemental UV-A wavelengths of 366 and 390 nm significantly increased the nitrate content of mustard microgreens at both irradiance durations (**Figure 3**). These results are consistent with those of other studies, where UV-A exposure has been shown to increase the nitrate level in lettuce (Voipio and Autio, 1995; Chang and Chang, 2014), basil, and beet microgreens (Brazaitytė et al., 2015). However, the maximum level of nitrate in mustard microgreens did not exceed 900 mg kg^–1^ and should not have a negative effect on human health. According to European Union regulations, the maximum limit of nitrate concentration in rucola (*Eruca vesicaria* L.) is 6,000–7,000 mg kg^–1^ (Commission Regulation (EU) No 1258/2011, 2011).

Mineral elements are important for plants as well as humans. They act as cofactors in vitamin, protein, and enzyme biosynthesis pathways and are essential for different metabolic processes (Sofo et al., 2016; Xiao et al., 2016). Since light is one of the main factors affecting the physiological processes in plants, it also affects mineral element uptake from the roots to shoots. Our experiments revealed that longer UV-A exposure (16 h) had the highest positive effect on the content of mineral elements in mustard microgreens. By contrast, shorter UV-A exposure (10 h) decreased the content of most macroelements and all microelements (**Table 2**, **Table 3**). Longer irradiance duration of supplemental 390-nm UV-A positively affected the content of P, Ca, and B, whereas 402-nm UV-A positively affected the content of P, Mg, Mn, Zn, and Cu. Mickens et al. (2019) reported contrasting results, where ASRM with UV-A light decreased the macroelement content of red pak choi. However, UV-A intensity in the ASRM (5 μmol m^–2^ s^–1^) was lower than our experiment (12.4 μmol m^–2^ s^–1^). This suggests that plants receiving a large amount of UV-A have a more intensive mineral uptake from roots to shoots, although further investigation is necessary. Additionally, as mentioned above, UV-A as well as blue light is perceived by the same photoreceptors; therefore, it can be assumed that the effect of UV-A on mineral elements is similar to that of blue light. Previous studies indicated that blue light had a significant effect on membrane potential and ion transport across membranes, which affects stomatal opening (Kinoshita et al., 2001; Babourina et al., 2002). It has also been shown that blue light increased the accumulation of macro- and microelements in plant tissues (Kopsell et al., 2014). However, whether UV-A triggers the same or different pathways as blue light needs further investigation.

## Conclusion

In summary, we determined the effects of different UV-A wavelengths and irradiance durations on mustard microgreens. Although UV-A did not affect biomass accumulation, longer wavelengths caused an increase in leaf area. Additionally, 402- nm UV-A at a longer duration (16 h) and 366 and 390-nm wavelengths at both irradiance durations (10 and 16 h) increased the accumulation of phenols and α-tocopherol. By contrast, lutein/zeaxanthin and β-carotene contents increased upon exposure of shorter UV-A wavelengths at 10-h irradiance as well as longer UV-A wavelengths at 16-h irradiance. The most positive effect on the accumulation of mineral elements, except Fe, was observed under the longer duration of UV-A irradiance at longer wavelengths. Based on the limited literature available on UV-A LED exposure and the results obtained in the current study, we suggest that properly composed UV-A LED parameters (wavelength, intensity, and irradiance duration) in LED lighting systems could improve the nutritional quality of leafy vegetables cultivated in CEA systems, without adverse effects on their growth.

## Data Availability Statement

All datasets generated for this study are included in the manuscript/**Supplementary Files**.

## Author Contributions

AB, AV, GS, and PD were involved in experimental design, performed the statistical data analyses, and wrote the manuscript. AB and JJ were responsible for conducting the experiments, and biometric and chlorophyll index measurements. VV-K performed mineral elements and nitrates analysis and interpreted the data, and wrote the methodical part of the manuscript. AV, GS, and JM performed nutritional quality analysis and interpreted the data. AN improved and maintained the lighting device. All authors approved the final manuscript.

## Funding

This project has received funding from the European Regional Development Fund (project No 01.2.2-LMT-K-718-01-0049) under a grant agreement with the Research Council of Lithuania (LMTLT).

## Conflict of Interest

The authors declare that the research was conducted in the absence of any commercial or financial relationships that could be construed as a potential conflict of interest.
